# Aberrant expression and subcellular localization of laminin 332 in oral squamous cell carcinoma: Implications for invasive front assessment

**DOI:** 10.4317/medoral.28040

**Published:** 2026-06-28

**Authors:** Nguyet Anh Thi Nguyen, Hong Thi Nguyen, Ngoc Hieu Nguyen, Thu Thao Thi Luu, Hai Ninh Truong

**Affiliations:** 1 University of Medicine and Pharmacy at Ho Chi Minh City, Ho Chi Minh City, Vietnam; 2 Nguyen Tat Thanh University, Ho Chi Minh City, Vietnam

**Keywords:** Laminin 332 (LM332), oral epithelial cancer, immunohistochemistry.

## Abstract

**Background:**

Laminin 332 (LM332) is a primary component of the epithelial BM. During malignant transformation, loss of continuity and cytoplasmic redistribution have been documented during tumor progression. This study aimed to evaluate LM332 expression at the basement membrane (BM) and in the cytoplasm of oral squamous cell carcinoma (OSCC) and to investigate its association with histopathological characteristics.

**Materials and Methods:**

A cross-sectional study was conducted on 130 patients utilizing immunohistochemical techniques to examine LM332 expression.

**Results:**

In OSCC, LM332 expression at the BM was frequently fragmented in 62% of cases and absent in 18%. Conversely, LM332 was significantly overexpressed in the cytoplasm, with 95% of cases exhibiting positive staining (≥1+ based on the semi-quantitative scoring system). A statistically significant difference was observed between LM332 expression and the histological grade of OSCC (p<0.05).

**Conclusions:**

LM332 immunohistochemical expression patterns may serve as an adjunctive marker reflecting histopathological progression in malignant oral epithelium.

## Introduction

Oral squamous cell carcinoma (OSCC) is a prevalent worldwide malignancy, with 377,713 new cases and 177,757 deaths reported in 2020 [1,2]. In Vietnam, the 2014 Ho Chi Minh City cancer registry recorded an incidence of 3.6 per 100,000 for oral and pharyngeal cancers. OSCC often presents at advanced stages due to local invasion and nodal metastasis, leading to poor survival rates and high treatment costs [3,4].

The BM, situated between the epithelium and connective tissue, is a thin yet resilient structure essential for regulating cellular activities [5]. As a dynamic barrier, it undergoes continuous remodeling through the turnover of glycoproteins, primarily mediated by laminins. Immunohistochemical assessment of BM components has proven valuable for cancer diagnosis and prognosis, given that the BM functions as both a structural scaffold and a critical barrier to malignant invasion and tumor dissemination. Laminins, large glycoproteins (400-900 kDa), participate in diverse cellular processes including adhesion, migration, proliferation, proteolysis, tumor growth, and metastasis [6]. In humans, 11 laminin genes have been identified, encoding five α-chains (LAMA1-5), three β-chains (LAMB1-3), and three γ-chains (LAMC1-3); LAMB4 has also been described but characterized as a pseudogene [7]. Each laminin forms an obligate heterotrimer consisting of one α, one β, and one γ chain, such as LM332 (α3β3γ2, LM332) [8].

In OSCC, LM332 is critically implicated in tumor progression. Transcriptome analyses have revealed LM332 overexpression, highlighting its association with invasive behavior and the risk of recurrence at surgical margins. Elevated LM332 expression is also associated with poor survival, supporting its prognostic relevance [9]. Immunohistochemical and cell biology studies consistently demonstrate strong LM332 expression in invasive regions, with levels correlating with tumor aggressiveness [10,11]. Moreover, *in vitro* experiments indicate that LM332 expressing oral cancer cells display enhanced invasiveness and migration compared with less aggressive cell lines, supporting their involvement in tumor progression [12].

## Materials and Methods

*Patient selection and inclusion criteria*

A total of 130 patients diagnosed with OSCC were enrolled in this study. All patients underwent surgical treatment at the Department of Head and Neck Surgery, Ho Chi Minh City Oncology Hospital, Vietnam. The study protocol was approved by the Ethics Committee in Biomedical Research at the University of Medicine and Pharmacy at Ho Chi Minh City, Vietnam. Ethical clearance was granted on September 22, 2014, under document number 216/DHHD-HD. Patients were included if they met the following criteria: Histologically confirmed OSCC according to the WHO Classification of Head and Neck Tumours; availability of complete clinicopathological data; and availability of representative formalin-fixed paraffin-embedded (FFPE) blocks containing both the tumor core and the invasive front. The exclusion criteria were: Prior receipt of radiotherapy, chemotherapy, or immunotherapy; recurrent OSCC; or biopsy samples with extensive necrosis or insufficient stromal tissue that precluded accurate assessment of basement membrane continuity. Clinical, pathological, and relevant risk factor data were retrospectively extracted from the patients’ medical records.  Data collection was conducted between January 2013 and December 2015, covering the patients’ diagnostic and treatment period.

*Immunohistochemistry*

Formalin-fixed paraffin-embedded (FFPE) tissues were sectioned at a thickness of 4 μm. Following deparaffinization and rehydration, antigen retrieval was performed in citrate buffer (pH 6.0) using a microwave oven (95°C for 20 min). Endogenous peroxidase activity was blocked with 3% hydrogen peroxide for 10 minutes. The sections were then incubated overnight at 4°C with the mouse monoclonal primary antibody against Laminin 332 (Clone P3E4, Santa Cruz Biotechnology, USA) at a dilution of 1:100. Signal detection was performed using the EnVision+ System-HRP Labelled Polymer (Dako) and 3,3'-diaminobenzidine (DAB) substrate. Sections were counterstained with Mayer’s hematoxylin. Normal oral mucosa with an intact basement membrane (BM) served as a positive control, whereas the primary antibody was replaced with phosphate-buffered saline (PBS) for negative controls. To ensure diagnostic objectivity and minimize bias, two experienced oral pathologists independently evaluated the slides in a blinded manner, without prior knowledge of the clinical data. Any scoring discrepancies were resolved by consensus using a multi-head microscope.

The expression of LM332 was categorized into two distinct patterns: BM Pattern: Classified as 'Continuous' (linear staining along the BM), 'Fragmented' (interrupted linear staining), or 'Absent' (a total absence of staining at the tumor-stroma interface); Cytoplasmic Pattern: Evaluated based on the percentage of positive tumor cells and staining intensity. For the cytoplasmic pattern, a semi-quantitative scoring system was applied: 0 (negative), 1+ (weak/focal), 2+ (moderate), and 3+ (strong/diffuse). Focal cytoplasmic accumulation was specifically analyzed at the invasive front of the tumor.

*Statistical Analysis*

Statistical analyses were performed using Stata 14.0 software. A 95% confidence level was set, and a statistical significance threshold was established at *p<*0.05. To evaluate the association between the LM332 expression patterns and clinicopathological characteristics, the Chi-squared test was employed for categorical variables when more than 80% of expected cell counts exceeded 5. Conversely, Fisher's exact test was applied when more than 20% of the cells had expected counts of 5 or less.

## Results

*Differential expression of LM332 in oral squamous cell carcinoma, premalignant lesions, and normal mucosa*

Among the 130 OSCC cases, 20% exhibited continuous LM332 staining along the basement membrane surrounding tumor nests, with immunoreactivity above or equal to 50% of the BM. 62% showed discontinuous BM staining, with less than 50% of the BM remaining immunoreactive (Table 1).

No significant associations were observed between this pattern and gender, age, tumor location, tumor size, lymph node status, or clinical stage (*p>*0.05). However, a statistically significant association was found with histological grade (*p<*0.05). The proportion of tumors with absent LM332 staining at the tumor-stroma interface increased progressively from well-differentiated to poorly differentiated OSCC.

*Comparison of BM LM332 expression among clinical and pathological subsets*

**Figure 1:**
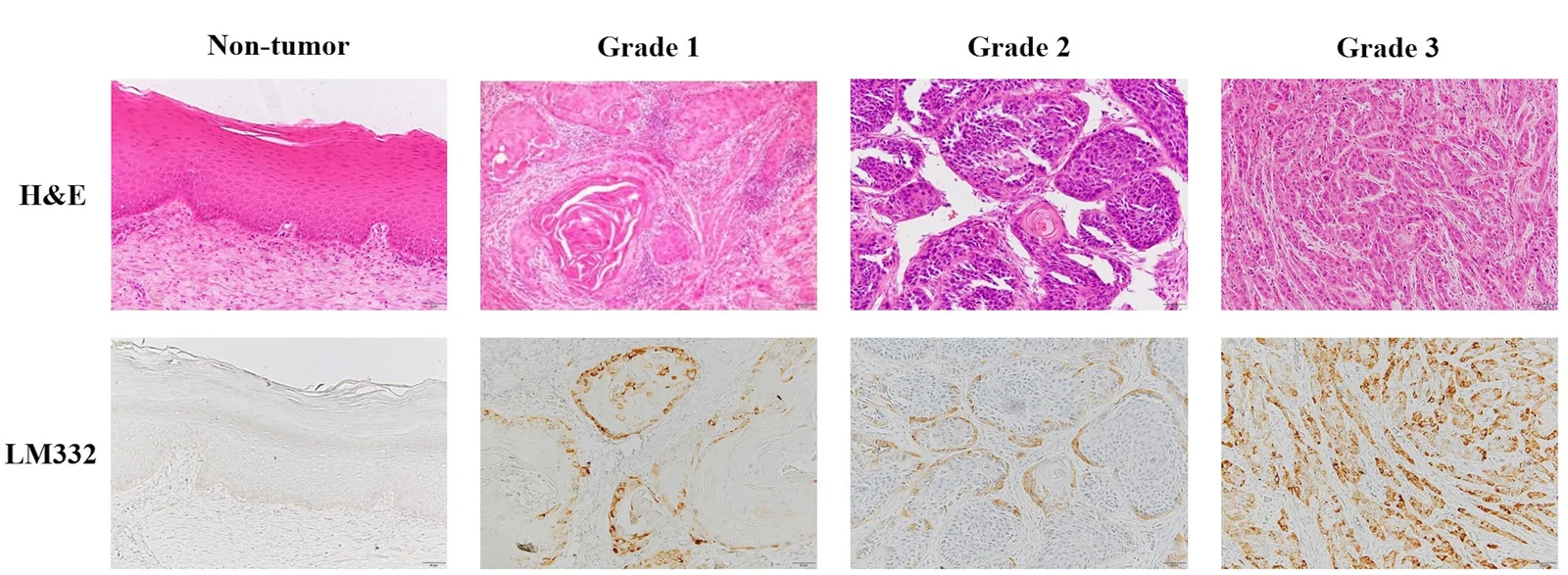
Fig. 1: Differential expression of LM332 in normal oral mucosa and OSCC.

No significant associations were observed between the BM LM332 staining pattern and patient gender, age, tumor location, primary tumor size, lymph node metastasis status, or clinical stage (*P>*0.05). However, a statistically significant association was found with histological grade (*p<*0.05). Although the majority of squamous cell carcinomas retained a discontinuous BM surrounding the invasive tumor nests (58.9% in grade 1; 67.2% in grade 2; 50% in grade 3) (Figure 1). The proportion of cases exhibiting a total absence of BM staining around the tumor nests increased progressively, rising from grade 1 (5.4%) to grade 2 (25%), and peaking at grade 3 (40%) (*p<*0.05) (Table 1).

*The relationship between cytoplasmic LM332 expression and the clinical and pathological features of cancer*

Cytoplasmic LM332 expression was observed in the majority of OSCC cases and was predominantly localized within the basal and suprabasal tumor cells, particularly at the invasive front of the tumor. No significant associations were found between cytoplasmic LM332 expression and patient age, gender, tumor location, primary tumor size, lymph node metastasis status, or clinical stage (*P>*0.05). However, there was a significant association with the histological grade of cancer. Furthermore, the proportion of cases with LM332 expression levels of grade 2 and 3 (>10%) increased progressively from grade 1 (64.3%) to grade 2 (70.3%), and was highest in grade 3 (90%). Conversely, the proportion of cases with expression levels of grade 0 and 1 (≤10%) decreased from grade 1 (35.7%) to grade 2 (29.7%), and was lowest in grade 3 (10%) (*p<*0.05) (Table 2).

## Discussion

In this study, we evaluated the expression patterns of LM332 in a cohort of 130 OSCC patients. Our results demonstrate a significant topographic shift of LM332 expression in OSCC. The fragmentation and eventual loss of LM332 in the BM signify proteolytic degradation of the extracellular matrix, a prerequisite for tumor invasion. In the context of invasive OSCC, the native basement membrane is frequently disrupted. Therefore, LM332 immunoreactivity outlining tumor cell nests at the tumor-stroma interface likely represents tumor-associated or partially reconstituted basement membrane (BM)-like structures rather than an intact physiological BM. This distinction is crucial for the accurate interpretation of immunohistochemical findings. The high frequency of fragmented or absent LM332 staining at the tumor-stroma interface observed in this study supports the concept of dynamic extracellular matrix remodeling during tumor progression. Proteolytic degradation of BM components, together with altered laminin synthesis by tumor cells, contributes to discontinuous or absent staining patterns.

Importantly, these alterations in LM332 expression were significantly associated with histological grade, suggesting that they reflect changes in tumor differentiation status. However, these findings should be interpreted as indicative of tumor biological behavior rather than as a direct measure of invasive capacity.

In parallel, the high prevalence of cytoplasmic LM332 (95% at level 1 and above) suggests that cancer cells at the invasive front actively synthesize this protein to facilitate cell migration and adhesion to the provisional matrix. This phenomenon, often linked to the accumulation of the LM322 γ2 chain monomer, serves as a hallmark of the invasive phenotype in oral malignancies and correlates with the higher histological grades observed in our cohort (*p<*0.05).

Immunohistochemical evaluation of BM components is valuable for cancer diagnosis and prognosis [13]. The BM, a dynamic barrier between epithelium and stroma, is essential for tissue organization, and its disruption, particularly via altered laminin expression and cytoplasmic localization in tumor cells, is linked to invasiveness and prognostic outcomes. In this study, regarding LM332 expression in the BM surrounding tumor cell nests, 80% of cases demonstrated BM defects, characterized by discontinuity or complete loss, while only 20% preserved a continuous BM. This distribution is consistent with the observations reported by Rahman [14].

The loss of laminin expression progressively increases from normal epithelium to dysplasia and carcinoma. In severe dysplasia or carcinoma *in situ*, the BM is still maintained, with thickened and thinned epithelial layers, displaying focal thickening or multilayering of the basement membrane [15]. These structural instabilities explain the finding of 104 cases with BM defects in the present study. LM332 expression reflects alterations in BM integrity during carcinogenesis and has been reported to be associated with tumor progression [16,17].

The frequency of BM discontinuity observed in OSCC in this study supports the findings of several previous studies [18], which noted loss of BM continuity around tumor nests. Furnes explained that when normal epithelium is replaced by cancer cells, the capacity for repair is diminished or defective, leading to BM discontinuity, which can be demonstrated through the immunoreactivity of laminin [19]. Similarly, it has been proposed that BM disruption in cancer results from altered synthesis and deposition of laminin by tumor cells [20].

The study found that cytoplasmic LM332 expression in OSCC was most commonly of moderate intensity (49%), followed by focal staining (25%), strong positivity (21%), and complete absence (5%). In comparison, Rahman reported that among 25 histologically diagnosed OSCC cases, 10 cases (40%) showed complete absence of laminin expression, with tumor cells infiltrating beyond the normal BM into the underlying tissue [14]. This 40% complete absence rate is higher than that observed in our cohort (5%); however, our 5% negative rate is consistent with the findings of Sakr *et al*. [21]. This loss of expression may be due to tumor cells lacking the ability to synthesize BM proteins or undergoing phenotypic changes toward malignancy. Notably, in Rahman’s study, only one case (5%) of OSCC retained laminin staining at the tumor-stroma interface, whereas our study observed a higher proportion (21%), indicating that certain normal functional characteristics of differentiated squamous epithelium were partially preserved.

Rani’s study was the first to describe the increased cytoplasmic LM332 expression in malignant cells [11], demonstrating the overexpression of LM332 exclusively in cancer tissue and not in dysplastic lesions. In contrast, other studies have focused on LM332 expression in the stroma [16,22]. The findings in our study align with Rani’s reports, showing that increased LM332 expression by immunohistochemistry was detected only in cases with clear malignant cell invasion.

Our analysis of laminin expression and BM integrity revealed intact, defective, or absent BM patterns across all levels of tissue differentiation. In well-differentiated tumors, LM332 expression with a discontinuous BM pattern accounted for 58.9%. This indicates that both tumor-adjacent cells and tumor cells still retain the ability to secrete laminin, consistent with similar descriptions in the literature [23]. In the poorly differentiated group, 10% of tumors still exhibited a continuous BM pattern. This contrasts with other studies, which reported that no high-grade malignant lesions exhibited a continuous BM. Instead, only 4 out of 12 lesions maintained an intact BM in 50% of cases [6,24]. This suggests that, in such cases, disruption of the BM surrounding tumor nests does not occur at every stage of tumor progression.

Thus, our findings demonstrate progressive alterations of BM integrity across different histological grades. The BM surrounding tumor cells becomes discontinuous during active invasion and is reconstituted during latent phases [6]. However, in the present analysis, invasive and latent stages may have overlapped. Maatta *et al*. noted that human carcinomas are often capable of synthesizing multiple laminin chains of different sizes, resulting in diverse laminin isoforms [25]. Furthermore, carcinomas express nearly all laminin chains within their BMs, suggesting that deposition of these macromolecules is not completely lost during tumor invasion. This may explain the observation of one case (10%) with a continuous BM and five cases (50%) with a discontinuous LM332 BM among the 10 high-grade malignant lesions in our study.

In the present study, cytoplasmic staining of LM332 was observed across all histological grades of OSCC, consistent with the findings of Mostafa *et al*. [26]. Complete negative staining for LM332 was detected in only 5.4% of cases, exclusively in grade 1 and grade 2 tumors, with no negative cases in grade 3. Statistical analysis showed that the frequency of LM332 expression increased with decreasing histological differentiation: 64.2% in well-differentiated OSCC, 70.3% in moderately differentiated OSCC, and 90% in poorly differentiated OSCC. Conversely, 35.7% of well-differentiated, 29.7% of moderately differentiated, and 10% of poorly differentiated OSCC cases showed absent or patchy cytoplasmic expression of LM332 in tumor cells. These results are comparable to those reported by Shruthy [18], who observed progressively increasing cytoplasmic laminin staining from well-differentiated carcinomas (30%) to moderately differentiated (80%) and highest in poorly differentiated cases (90%).

The study results also did not show a significant association between cytoplasmic expression of LM332 and the clinical or histopathological parameters of OSCC (except for tumor histological grade). This finding is consistent with Gasparoni’s report [27], which also found no correlation with the clinical or histopathological features of the tumor. This study also sampled from multiple sites within the oral cavity, and the author suggested that LM332 should be considered a negative prognostic factor in OSCC.

Interestingly, no significant association was found between LM332 expression and lymph node metastasis. This finding may be attributed to several factors. First, the relatively small proportion of advanced-stage tumors in our cohort may have limited the statistical power to detect such correlations. Second, LM332 expression may primarily reflect local invasive remodeling at the tumor-stroma interface rather than systemic metastatic dissemination. Finally, the cross-sectional design of this study precludes the evaluation of dynamic metastatic progression over time.

Despite the significant findings, this study has limitations. As a cross-sectional study, it precludes the provision of longitudinal data correlating LM332 expression with long-term survival rates or regional recurrence. Furthermore, tumor budding, a recently recognized prognostic marker in OSCC, was not evaluated in the present study due to the limited availability in retrospective slides. Future studies incorporating tumor budding assessment may further clarify the potential prognostic value of LM332 expression [28]. Additionally, while IHC provides spatial localization, molecular assays such as Western blot or RT-PCR could further quantify the specific laminin chains involved in the invasion process.

## Conclusions

LM332 expression patterns in OSCC vary with histopathological grade. The observed variations in basement membrane integrity and cytoplasmic localization may reflect structural alterations during tumor development. Further studies are needed to clarify the clinical and prognostic significance of these findings. 

## Figures and Tables

**Table 1:** Table 1: Association between BM expression of LM332 and the clinical and pathological features of oral mucosal cancer.

**Characteristics**	**Expression of LM332 at the BM**				**P**
	**Total** N (%)	**Absent BM** **N = 23**	**Continuous BM** **N = 26**	**Discontinuous BM** **N = 81**	
**Gender**					0.233^a^
Female	48 (36.9)	12 (25.0)	8 (16.7)	28 (58.3)	
Male	82 (63.1)	11 (13.4)	18 (22.0)	53 (64.6)	
**Age**					0.368^b^
<40	13 (10.0)	3 (23.1)	4 (30.8)	6 (46.2)	
≥40	117 (90.0)	20 (17.1)	22 (18.8)	75 (64.1)	
Smoking					0.265^a^
Yes	47 (36.2)	5 (10.6)	11 (23.4)	31 (66.0)	
No	83 (63.8)	18 (21.7)	15 (18.1)	50 (60.2)	
Betel chewing					0.999^b^
Yes	4 (3.1)	-	1 (25.0)	3 (75.0)	
No	126 (96.9)	23 (18.3)	25 (19.8)	78 (61.9)	
Alcohol consumption					0.370^b^
Yes	37 (28.5)	4 (10.8)	9 (24.3)	24 (64.9)	
No	93 (71.5)	19 (20.4)	17 (18.3)	57 (61.3)	
Tumor location					0.273^b^
Tongue	64 (49.2)	13 (20.3)	17 (26.6)	34 (53.1)	
Floor of mouth	23 (17.7)	3 (13.0)	1 (4.3)	19 (82.6)	
Gingiva	16 (12.3)	4 (25.0)	4 (25.0)	8 (50.0)	
Hard palate	3 (2.3)	-	-	3 (100.0)	
Buccal mucosa	8 (6.2)	2 (25.0)	1 (12.5)	5 (62.5)	
Lips	16 (12.3)	1 (6.3)	3 (18.8)	12 (75.0)	
Primary tumor					0.452^b^
T1	28 (21.5)	6 (21.4)	5 (17.9)	17 (60.7)	
T2	64 (49.2)	7 (10.9)	13 (20.3)	44 (68.8)	
T3	15 (11.5)	5 (33.3)	3 (20.0)	7 (46.7)	
T4	23 (17.7)	5 (21.7)	5 (21.7)	13 (56.5)	
Nodal metastasis					0.797^a^
N0	91 (70.0)	17 (18.7)	19 (20.9)	55 (60.4)	
N1-2-3	39 (30.0)	6 (15.4)	7 (17.9)	26 (66.7)	
Stage of cancer					0.939^a^
1	25 (19.2)	6 (24.0)	5 (20.0)	14 (56.0)	
2	47 (36.2)	6 (12.8)	9 (19.1)	32 (68.1)	
3	25 (19.2)	5 (20.0)	5 (20.0)	15 (60.0)	
4	33 (25.4)	6 (18.2)	7 (21.2)	20 (60.6)	
Histologic grade					<0.001 ^b^
Grade 1	56 (43.1)	3 (5.4)	20 (35.7)	33 (58.9)	
Grade 2	64 (49.2)	16 (25.0)	5 (7.8)	43 (67.2)	
Grade 3	10 (7.7)	4 (40.0)	1 (10.0)	5 (50.0)	

^a^ Chi-square test; ^b^ Fisher's exact test.

**Table 2:** Table 2: Association between cytoplasmic expression of LM332 and the clinical and pathological features of oral mucosal cancer.

**Oral mucosal cancer**	**Cytoplasmic LM332**					**P**
	**Total N** **(%)**	**Grade 0** 0% N = 7	**Grade 1** 1-10% N = 33	**Grade 2** 11-50% N = 63	**Grade 3** > 50% N = 27	
**Gender**						0.233
Female	48 (36.9)	5 (10.4)	13 (27.1)	20 (41.7)	10 (20.8)	
Male	82 (63.1)	2 (2.4)	20 (24.4)	43 (52.4)	17 (20.7)	
**Age**						0.768
<40	13 (10.0)	1 (7.7)	2 (15.4)	7 (53.8)	3 (23.1)	
≥40	117 (90)	6 (5.1)	31 (26.5)	56 (47.9)	24 (20.5)	
Smoking						0.594
Yes	47 (36.2)	2 (4.3)	15 (31.9)	20 (42.6)	10 (21.3)	
No	83 (63.8)	5 (6.0)	18 (21.7)	43 (51.8)	17 (20.5)	
Betel chewing						0.637
Yes	4 (3.1)	-	2 (50.0)	2 (50.0)	-	
No	126 (96.9)	7 (5.6)	31 (24.6)	61 (48.4)	27 (21.4)	
Alcohol consumption						0.624
Yes	37 (28.5)	1 (2.7)	8 (21.6)	18 (48.6)	10 (27.0)	
No	93 (71.5)	6 (6.5)	25 (26.9)	45 (48.4)	17 (18.3)	
Tumor location						0.234
Tongue	64 (49.2)	1 (1.6)	15 (23.4)	34 (53.1)	14 (21.9)	
Floor of mouth	23 (17.7)	-	5 (21.7)	13 (56.5)	5 (21.7)	
Gingiva	16 (12.3)	3 (18.8)	4 (25.0)	6 (37.5)	3 (18.8)	
Hard palate	3 (2.3)	1 (33.3)	1 (33.3)	-	1 (33.3)	
Buccal mucosa	8 (6.2)	1 (12.5)	2 (25.0)	4 (50.0)	1 (12.5)	
Lips	16 (12.3)	1 (6.3)	6 (37.5)	6 (37.5)	3 (18.8)	
Primary tumor						0.760
T1	28 (21.5)	3 (10.7)	7 (25.0)	11 (39.3)	7 (25.0)	
T2	64 (49.2)	3 (4.7)	14 (21.9)	33 (51.6)	14 (21.9)	
T3	15 (11.5)	-	5 (33.3)	9 (60.0)	1 (6.7)	
T4	23 (17.7)	1 (4.3)	7 (30.4)	10 (43.5)	5 (21.7)	
Nodal metastasis						0.319
N0	91 (70.0)	7 (7.7)	23 (25.3)	44 (48.4)	17 (18.7)	
N1-2-3	39 (30.0)	-	10 (25.6)	19 (48.7)	10 (25.6)	
Stage of cancer						0.722
1	25 (19.2)	3 (12.0)	5 (20.0)	10 (40.0)	7 (28.0)	
2	47 (36.2)	3 (6.4)	12 (25.5)	25 (53.2)	7 (14.9)	
3	25 (19.2)	-	8 (32.0)	12 (48.0)	5 (20.0)	
4	33 (25.4)	1 (3.0)	8 (24.2)	16 (48.5)	8 (24.2)	
Histologic grade						0.048
Grade 1	56 (43.1)	2 (3.6)	18 (32.1)	31 (55.4)	5 (8.9)	
Grade 2	64 (49.2)	5 (7.8)	14 (21.9)	27 (42.2)	18 (28.1)	
Grade 3	10 (7.7)	-	1 (10.0)	5 (50.0)	4 (40.0)	

Fisher's exact test.

## Data Availability

None.
